# Psychometric properties of the Chinese Family Assessment Instrument: evidence from mainland China

**DOI:** 10.3389/fpsyg.2023.1290224

**Published:** 2023-12-11

**Authors:** Daniel T. L. Shek, Kim Hung Leung, Xiang Li, Diya Dou

**Affiliations:** Department of Applied Social Sciences, The Hong Kong Polytechnic University, Hong Kong, Hong Kong SAR, China

**Keywords:** Chinese Family Assessment Instrument, psychometric properties, China, family functioning, measurement invariance

## Abstract

Regarding the assessment of family functioning in Chinese people, there are several research gaps. First, although there are some instruments in the field, there are very few validated instruments. Second, while some translated measures have been developed, there are very few assessment tools based on indigenous Chinese concepts. Third, compared to Hong Kong, research on family assessment is relatively inactive in mainland China. Fourth, there are very few family assessment tools to assess perceived family functioning in older children and early adolescents. Fifth, few studies used large samples to validate family assessment tools. Sixth, researchers seldom utilized longitudinal data to examine the psychometric properties of family assessment tools. Finally, few studies have examined factorial validity across samples and time to demonstrate the stability of Chinese family assessment measures. In Hong Kong, based on focus group data (i.e., indigenous concepts of family functioning) and an integration with the family science literature, we have developed the Chinese Family Assessment Instrument (C-FAI) to assess perceived family functioning according to the perception of adolescents. Results showed that the C-FAI possessed good reliability and validity. Specifically, five dimensions of the measure (mutuality, communication, conflict, parental concern and parental control) were supported via exploratory factor analysis and confirmatory factor analysis. Convergent validity and reliability of the C-FAI were illustrated. To understand the psychometric properties of the C-FAI in mainland China, we collected three waves of data from students in the period of preadolescence and early adolescence in mainland China (*N* = 3,732). Based on the data, we examined the psychometric properties of the measure, particularly factor invariance in different samples and at different times. Confirmatory factor analysis provided support for the five dimensions in C-FAI, including factorial invariance in terms of configuration, factor loading, intercepts, and over time. There was evidence for convergent validity and discriminant validity of the measure. Finally, reliability analyses showed that the total C-FAI scale and its subscales are internally consistent. The present findings suggest that family researchers and practitioners can use the C-FAI to objectively assess perceived family functioning in preadolescence and early adolescence in different Chinese communities.

## Introduction

The concept of family functioning can be viewed as the general quality of the family environment and the relationships among its members (Folk et al., 2020). Different family theories, such as Beavers system theory (Beavers and Hampson, 2000), McMaster family functioning mode theory (Miller et al., 2000) and family therapy theories (Alexander and Parsons, 1982; Minuchin, 2012) have proposed different but conceptually related dimensions of family functioning. For example, Beavers system theory proposed six dimensions of family functioning, including family structure, mythology, goal-directed negotiation, autonomy, family affect, and global health pathology (Beavers and Hampson, 2000). Besides, the McMaster family functioning model theory proposes six dimensions of family functioning, such as effective communication, clear family roles, and appropriate affective responses. Studies have revealed the positive impact of positive family functioning on the developmental outcomes of children and adolescents such as engagement in learning, happiness, mental health, and proper behaviors (Izzo et al., 2022; Tamayo-Aguledo et al., 2022; Peng S. et al., 2023; Qi et al., 2023).

To assess family functioning, researchers have adopted different assessment methods such as direct observation (Giusto et al., 2019), interviews (Sumari et al., 2020), and self-reported instruments like Olson’s (2000) Family Adaptability and Cohesion Evaluation and Epstein et al.’s (1983) McMaster Family Assessment Device. In fact, self-reported family functioning scales are commonly utilized to examine the perceived family functioning of people (Cong et al., 2022). As such, the development of family functioning measures with sound psychometric properties is of paramount importance for clinical and research purposes. However, most of the studies are WEIRD studies, with data collected from Western, educated, industrialized, rich, and democratic societies.

As most of the family functioning measures have been developed in the West, researchers have translated and adapted these measures into their local languages like Portuguese and German (e.g., Beierlein et al., 2017; Almeida et al., 2020). Nevertheless, other researchers have challenged cross-cultural adaptation of these measures because of cross-cultural differences, such as differences in individualistic versus collectivistic values in different cultures. In Sumari et al.’s (2021) study, the authors found that some factors of their indigenous family functioning scale were the same as those identified in the Family Assessment Device and Family Environment Scale. However, these factors had different meanings based on Malaysian local cultural understanding and interpretations. For instance, the communication and cohesion factors have the elements of courtesy and tolerance, respectively, and this reflects the importance of the preservation of family harmony in Malaysian collectivistic culture. Besides, other researchers have constructed indigenous measures to assess the perceptions of family functioning in their own countries, such as the Japanese version of Survey of Family Environment (Hohashi and Honda, 2012) and Korean version of Family Dynamic Environment Scale (Kim and Kim, 2007).

With specific reference to mainland China, there is rapid growth of family interventions in mainland China. The increase in the research on the importance of improving family functions for parents and children (e.g., Mao et al., 2019; Zhang et al., 2020; Lee et al., 2023) has called for the development of validated assessment tools to objectively examine family functioning in mainland China (Siu and Shek, 2005; Shek, 2006). Nevertheless, there are very few holistic, validated family functioning instruments despite the fact that Chinese people constitute roughly one-fifth of the world population. After checking with the PsycINFO database using “family assessment” in Abstract in November 2023, we found 27,967 records. However, we found only 32 records of family assessment using “family assessment” and “mainland China” and 121 records of family assessment using “family assessment” and “Hong Kong.” Besides, while there are validated translated instruments such as the Chinese version of Family Assessment Device (e.g., Wong et al., 2022), some important indigenous concepts of Chinese family functioning such as mutuality and avoidance of family conflict are lacking. Besides, although there are some existing Chinese family assessment tools, most of them are not comprehensive and only assess either family interaction (e.g., Wu et al., 2017) or parenting of Chinese families (e.g., Zhao et al., 2023). Furthermore, few studies have examined factorial validity of the different family functioning measures in China (Cheng et al., 2011; Zheng and Yang, 2022).

Responding to this gap, based on focus group data (i.e., indigenous concepts of family functioning) and integration with the family science literature, Shek (2002) developed the Chinese Family Assessment Instrument (C-FAI) to assess the perceived family functioning of Hong Kong adolescents. Specifically, the data gathered from focus groups with adolescents and their parents illustrated that the absence of conflict, family harmony, mutuality, sense of belonging, and good parent–child relationships were regarded as vital elements of a healthy family, whereas emotional expression and communication were least emphasized as important constituents of an optimal family. Past research has revealed that the C-FAI possesses good reliability and validity (Siu and Shek, 2005; Shek and Ma, 2010). In particular, the five dimensions of the C-FAI (mutuality, communication, conflict and harmony, parental concern and parental control) were validated by exploratory factor analysis and confirmatory factor analysis. There was also support for its convergent validity and reliability. However, the supporting evidence was confined to Hong Kong.

In view of cultural disparities between Hong Kong and mainland China, the applicability of the C-FAI to assess the perceived family functioning of adolescents in mainland China deserves further exploration. Under the principle of “one country, two systems,” Hong Kong does not possess the same economic and social systems as those in mainland China. For example, children acting in a non-filial manner will be publicly sanctioned in mainland China. Substance abuse in young people is also unique in Hong Kong (Shek, 2007). Besides, there are other differences between Hong Kong and mainland China, including (a) Hong Kong is more individualistic whereas mainland China is more collectivistic; (b) Hong Kong is a Capitalistic society whereas mainland China is a Socialist society with Chinese characteristics; (c) mainland China is still more susceptible to traditional Chinese values (e.g., Lunar New Year holidays). As such, exploration of the psychometric properties of the C-FAI, which was originally developed and validated using Hong Kong adolescents as the sample, is warranted for preadolescents and adolescents in mainland China.

Besides, there are several gaps in the existing literature in this field. First, validated family functioning measures in mainland China are very limited. Second, as mentioned above, compared to Hong Kong, research on family assessment is relatively inactive in mainland China. Third, there are very few family assessment tools to examine perceived family functioning in children in preadolescence and adolescence. As adolescents may have tense relationships with parents during puberty, understanding their perceived family functioning is important. Fourth, very few studies have adopted longitudinal research design with a large sample size to examine the psychometric properties of family functioning assessment tools. Fifth, few studies have investigated factorial validity across samples and time to demonstrate the stability of family assessment measures. In response to these research gaps, we asked several research questions in the present study based on students in preadolescence and early adolescence in mainland China:

*Research Question 1*: What are the dimensions underlying the C-FAI based on the responses of participants in preadolescence and adolescence? With reference to previous findings (Siu and Shek, 2005; Shek and Ma, 2010), we expected that the five-factor structure of the C-FAI would be supported (Hypothesis 1).

*Research Question 2*: Are the dimensions underlying the C-FAI invariant across random sub-samples? Based on Shek and Ma’s (2010) study, we hypothesized that the five-factor structure of the C-FAI would be invariant across random sub-samples (Hypothesis 2).

*Research Question 3*: Are the dimensions underlying the C-FAI invariant across time? We expected that the factor structure of the C-FAI would be invariant across time (Hypothesis 3).

*Research Question 4*: Is there support for the convergent validity of the C-FAI? Based on previous studies (e.g., Schumm et al., 1986; Shek et al., 1993; Gaspar et al., 2022), we expected that C-FAI scores would be positively related to measures of family support (Hypothesis 4).

*Research Question 5*: Is there support for the discriminant validity of the C-FAI? Drawing upon the practice of previous studies (Schumm et al., 1986; Shek et al., 1993), we expected that C-FAI scores would not be strongly correlated with the measures that are theoretically unrelated to family functioning (Hypothesis 5).

*Research Question 6*: What is the reliability of the C-FAI total and subscale measures? We expected that the total scale and subscales of the C-FAI would have acceptable reliability (Hypothesis 6).

## Materials and methods

### Participants and procedures

In this study, we conducted a 3-wave longitudinal research on the psychosocial adjustment of Chinese preadolescents and adolescents with data gathered at three different time points: a baseline (Wave 1), six months later (Wave 2), and one and a half years later (Wave 3) from the baseline (e.g., Dou et al., 2023; Peng L.-L. et al., 2023). In 2020, there were 623 elementary schools, 317 junior secondary schools, and 156 schools admitting both elementary and junior secondary students in Chengdu. All of them were public schools. Prior to the onset of the COVID-19 pandemic (Wave 1), a cluster sampling method was used to select five schools (one elementary school, one junior secondary school, and three admitting both elementary and junior secondary students) to participate in this study. Among these participating schools, two were situated in southern suburban areas, two were in northern suburban areas, and one was in the downtown area. In the scientific literature, there are studies in which data from elementary and secondary school students are collected (Chai et al., 2022; Obregón-Cuesta et al., 2022).

In sum, a total of 11,154 students from five selected schools participated in this study. Among them, 3,019 students completed the survey at either one wave, 2,008 students completed the survey at either two waves, and 6,127 students completed the survey at all three waves. Students were asked to answer an identical questionnaire containing a Chinese Family Assessment Instrument in class during the survey. For primary school students, the questions on the questionnaire were read aloud to the students, item by item, by the class teacher in each class. This practice is commonly used in similar studies in the field (Miller and Meece, 1997; Stutz et al., 2017). As such, the class teacher could help clarify any misunderstandings when asked questions by students. For high school students, students read the questions and responded to the questions on their own. Before starting the survey, we got consent to take part in the survey from parents and students in addition to ethics permission for research from Sichuan University. Moreover, some vital principles such as anonymity and voluntary participation were told to students. After the survey, students’ data at 3 waves were matched.

To understand the research questions for students in the period of preadolescence and early adolescence, we primarily examined the responses given by students aged 10 and above (e.g., Larson, 1997; McMakin and Alfano, 2015). In the matched sample aged 10 and above (*N* = 3,732), there were 1,938 primary school students at Wave 1 (51.8% males and 48.2% females; average age was 10.7 ± 0.72 years old; 99.1% Hans; 31.3% students have no siblings; average family monthly income was 118,773 CNY; 12.0% fathers and 10.5% mothers possess “university and above” as their highest educational level). There were 1,794 high school students at Wave 1 (49.4% males and 50.6% females; average age was 12.8 ± 0.76 years old; 99.2% Hans; 34.3% students have no siblings; average family monthly income was 181,531 CNY; 14.0% fathers and 11.8% mothers possess “university and above” as their highest educational level).

### Instrument

The students responded to a questionnaire assessing psychosocial adjustment in children and adolescents. It contains a 33-item Chinese Family Assessment Instrument (C-FAI) which has been employed to investigate the perceived family functioning of Chinese adolescents (Shek, 2002). It has five dimensions, including mutuality (12 items, e.g., “family members understand each other”), communication (9 items, e.g., “family members are cohesive”), harmony and conflict (6 items, e.g., “poor marital relationship of parents”), parental concern (3 items, e.g., “parents take care of their children”), and parental control (3 items, e.g., “parental control too harsh”). These five dimensions encompass the primary characteristics of positive family functioning in Chinese families, involving absence of conflict, mutuality, and effective communication among family members, in addition to favorable parent–child and spousal relationships. Students’ responses were assessed using a 5-point scale (1 = most similar, 5 = most dissimilar). All positively worded items were reverse coded. As such, an item score and the level of functioning of Chinese families was positively correlated. C-FAI has been found to be a valid and reliable tool for assessing family functioning in past studies using Hong Kong adolescents (e.g., Shek and Ma, 2010; Yu and Shek, 2013).

Besides the Chinese Family Assessment Scale, three additional items were employed to evaluate the convergent validity of the C-FAI: (a) mutual support among family members (“family members mutually support each other”); (b) degree of understanding of family members regarding the situations of each other (“family members know to understand the situations of each other”), and (c) relationship between the participant and his/her caregivers (“Is the relationship between you and your caregivers good?”). Students were asked to respond to the first two questions along a 6-point scale (1 = strongly disagree, 6 = strongly agree) and along a 10-point scale (1 = very worse, 10 = very well) for the last question.

Moreover, three additional items theoretically unrelated to family functioning were added to assess the discriminant validity of the C-FAI involving the items measuring the amount of time for sleeping (“What is your daily amount of sleeping time?”) and doing exercise (“What is your daily amount of time to do exercise”), and the amount of sweet drink students take in per week/month (“On average, how much sweet drink do you take in per week/month?). In the literature (e.g., Shek et al., 1993; Armenta et al., 2013; Tsukayama et al., 2013), researchers have used this approach to assess the discriminant validity of a measurement instrument.

### Data analysis

In this study, we performed confirmatory factor analysis (CFA) and measurement invariance (MI) tests to assess the factorial validity, convergent validity, discriminant validity, and reliability of the C-FAI, and its stability across groups and over time. CFA and MI tests were conducted using structural equation modeling techniques via Lisrel 8.54. Parameters were estimated by utilizing maximum likelihood estimation (ML) and robust maximum estimation (RML) methods. RML was chosen because it could reduce standard errors of the estimates caused by the violation of multivariate normality of the data. Convergent and discriminant validity of the C-FAI were assessed using Pearson correlation with the aid of SPSS 26.0.

The present investigation implemented five sequential steps. First, CFA was performed to assess the factor structure of the C-FAI using three waves of data (Wave 1 to Wave 3) individually. As stated by Brown (2006), the factor model of the C-FAI fits the data adequately when the values of the standardized root-mean-square residual and the root-mean-square error of approximation are less than 0.08 (MacCallum et al., 1996; Hu and Bentler, 1999), and the values of the non-normed fit index (NNFI) and the comparative fit index (CFI) are more than 0.90 (Bentler and Bonett, 1980).

Second, after identifying the factor structure of the C-FAI and establishing its factorial validity, we assessed the stability of the factor structure of the C-FAI across groups. Initially, the total sample at each wave was randomly divided into two subsamples based on cases. Multigroup confirmatory factor analysis (MCFA) was then used to assess the measurement invariance of the C-FAI across subsamples at each wave. Following the steps outlined by Dimitrov (2010), the levels of measurement invariance were assessed in the following order: separate groups, configural invariance, weak measurement invariance, strong measurement invariance, and strict measurement invariance. These steps are commonly followed when testing the measurement invariance of a scale (e.g., Castillo et al., 2015; Carr et al., 2017). Hence, a series of models ranging from least restrictive to most restrictive models were compared.

At the beginning, a five-factor model of the C-FAI was assessed separately for each group. Then, the five-factor structure of the C-FAI was evaluated simultaneously across groups to establish configural invariance in the analysis. The models were specified with no restriction in factor loadings, intercepts and uniqueness of the corresponding indicators between groups. Afterwards, weak measurement invariance was examined with the same models of configural invariance except the equality of factor loadings was imposed between the corresponding indicators of both groups. Later, strong measurement invariance was investigated with the same models of weak measurement invariance except the equalities of factor loadings and intercepts were imposed between the corresponding indicators of both groups. Finally, strict measurement invariance was examined with the same models of strong measurement invariance except the equalities of factor loadings, intercepts and uniqueness were imposed between the corresponding indicators of both groups. After establishing measurement invariance of the C-FAI, structural invariance of the 5-factor correlated model of the C-FAI was further explored by testing invariance in factor variances and factor covariances of the C-FAI model. Invariance in factor variances was examined with the same models of strict measurement invariance except the equalities of factor loadings, intercepts, and uniqueness were imposed between the corresponding indicators, and the equality of variances between corresponding factors was imposed between corresponding factors of both groups. Furthermore, invariance in factor covariances was assessed with the same models of factor variances invariance except the equalities of factor loadings, intercepts, and uniqueness were imposed between the corresponding indicators, and the equalities of variances and covariances were imposed between corresponding factors of both groups. For each form of factorial invariance, the model was compared with the model that preceded it.

As chi-square difference tests tend to reject the null hypothesis of no difference between two nested models in large samples even though the difference is trivial (Cheung and Rensvold, 2002), changes in CFI and RMSEA values were also commonly used to assess model fit for the factorial invariance of the C-FAI (Vandenberg and Lance, 2000). An acceptable model fit for more restrictive invariant models is based on the change in CFI value that is not more than 0.002 (Little, 2013), and the change in RMSEA value that is not more than 0.01 (Chen, 2007).

Third, after confirming the stability of the factor structure of the C-FAI across groups, we further tested whether the factor structures were stable across time. Identical factor analytic procedures and criteria for the fit of invariant nested models mentioned above were conducted to assess the stability of the factor structure across three waves of data (Wave 1 to Wave 3), with autocorrelation of uniqueness specified among same observable indicators in Wave 1, Wave 2 and Wave 3.

Fourth, apart from investigating the factorial validity and invariant properties of the C-FAI, we also assessed the convergent and discriminant validity of the C-FAI. The convergent validity of the C-FAI is demonstrated when the “average variance extracted” (AVE) for each factor at all waves was more than 0.50 (Hamid et al., 2017). It means that each construct explained more than 50% of the total variance in their respective indicators, and hence the convergent validity of the 5-factor structure of the C-FAI was supported. Besides, the convergent validity of the C-FAI was illustrated when the total score of the C-FAI is correlated significantly and substantially with the scores of three conceptually related items in the questionnaire, including (1) there is mutual support among family members, (2) family members know to understand the situations of each other, and (3) relationship between you and caregivers. On the other hand, the discriminant validity of the C-FAI was illustrated when the total score of C-FAI did not show any substantial correlation with those of items unrelated to the measurement of family constructs, such as the items tapping the amount of time for sleep and doing exercise, and the amount of sweet drink the participants take in per week and month. This approach was adopted in previous studies to examine the convergent and discriminant validity of a measure (e.g., Shek et al., 1993; Armenta et al., 2013; Tsukayama et al., 2013).

Lastly, we examined the reliability of the C-FAI using composite reliability, in which the acceptable value for it is 0.70 and above (Raykov, 2004). Moreover, we utilized Cronbach’s alphas and mean inter-item correlations of the C-FAI to further examine the internal consistency reliability of the subscales and the total scale of the C-FAI (see Schmitt, 1996). A value of Cronbach’s alpha greater than 0.7 indicates acceptable reliability, while the value of mean inter-item correlations in-between 0.3 to 0.7 illustrates adequate internal consistency of the scale (Lin et al., 2009).

## Results

### Descriptive statistics

Means, standard deviations, skewness, and kurtosis of each item of the C-FAI were assessed. The range of mean and standard deviation was 3.44–4.49 and 0.94–1.51, respectively. All items were normally distributed because the absolute values of univariate skewness (ranging from 0.43 to 2.00) and kurtosis (ranging from 1.26 to 3.76) values were not more than 2 and 7, respectively.

### Factorial validity of C-FAI

Table 1 summarizes the results of CFA according to the sample at Wave 1, Wave 2, and Wave 3. The findings of this study illustrated that the five-factor correlated model of C-FAI, with four error covariances fitted the data of each wave adequately (Wave 1: SBχ^2^ = 18,464, df = 481, *p* < 0.001, NNFI = 0.94, CFI = 0.94, RMSEA = 0.100, SRMR = 0.105; Wave 2: SBχ^2^ = 10,303, df = 481, *p* < 0.001, NNFI = 0.96, CFI = 0.96, RMSEA = 0.074, SRMR = 0.103; Wave 3: SBχ^2^ = 21,044, df = 481, *p* < 0.001, NNFI = 0.94, CFI = 0.95, RMSEA = 0.107, SRMR = 0.123). Nevertheless, it is noteworthy that the RMSEA and SRMR values in the model of each wave represented fair fit only, although NNFI and CFI indices illustrated good fit. Apart from two items (item 14 and item 23) which had the loadings less than 0.34, factor loadings of all other items were higher than 0.40 and significant at 0.05 level. As such, Hypothesis 1 was supported.

**Table 1:** Results of CFA of the five-factor correlated model of the C-FAI at Wave 1, Wave 2, and Wave 3.

Fit indices of the C-FAI model		Wave 1	Wave 2	Wave 3
SBχ^2^		18,464	10,303	21,044
df		481	481	481
*value of p*		<0.001	<0.001	<0.001
NNFI		0.94	0.96	0.94
CFI		0.94	0.96	0.95
RMSEA		0.100	0.074	0.107
SRMR		0.105	0.103	0.123
Standardized factor loadings				
Factors	Item number and content			
Mutuality	1. Family members support each other	0.77	0.81	0.85
	2. Family members love each other	0.79	0.84	0.88
	4. Family members care about each other	0.80	0.85	0.87
	5. Family members mutually consider each other	0.85	0.87	0.91
	6. Family members understand each other	0.82	0.85	0.88
	15. Family members get along well	0.64	0.82	0.82
	17. Family members have good relationship with each other	0.72	0.83	0.74
	18. Family members tolerate each other	0.63	0.67	0.65
	19. Family members are patient with each other	0.64	0.72	0.68
	20. Family members accommodate each other	0.55	0.62	0.62
	21. Family members trust each other	0.74	0.84	0.77
	32. Children are filial	0.56	0.60	0.58
Communication	7. Family members talk to each other	0.81	0.83	0.85
	8. Family members frequently arrange family activities	0.65	0.70	0.72
	9. Family members are cohesive	0.87	0.88	0.91
	10. Family members enjoy getting together	0.83	0.83	0.87
	11. Not many barriers among family members	0.71	0.73	0.78
	25. Parents know children’s needs	0.57	0.69	0.65
	26. Parents understand children’s mind	0.62	0.70	0.66
	27. Parents often talk to their children	0.62	0.71	0.67
	28. Parents share children’s concerns	0.57	0.65	0.62
Harmony and conflict	3. Family members do not mutually concern with each other	0.52	0.63	0.63
	12. Much friction among family members	0.59	0.60	0.61
	13. Frequent fighting among family members	0.71	0.69	0.62
	14. Not many quarrels among family members	0.25	0.26	0.31
	16. Lack of harmony among family members	0.69	0.74	0.73
	33. Poor marital relationship of parents	0.48	0.58	0.58
Parental concern	22. Parents love their children	0.86	0.85	0.85
	23. Parents do not care about their children	0.33	0.29	0.33
	24. Parents take care of their children	0.86	0.87	0.88
Parental control	29. Parents scold and beat children	0.80	0.82	0.85
	30. Parents force children to do things	0.79	0.81	0.81
	31. Parental control too harsh	0.68	0.72	0.70

AVE for the total scale and each factor, and inter-factor correlations																		
	Wave 1						Wave 2						Wave 3					
Factors	(1)	(2)	(3)	(4)	(5)	AVE	(1)	(2)	(3)	(4)	(5)	AVE	(1)	(2)	(3)	(4)	(5)	AVE
1. MU	1.0					0.51	1.0					0.61	1.0					0.61
2. COM	0.94	1.0				0.49	0.95	1.0				0.56	0.96	1.0				0.57
3. HC	0.49	0.45	1.0			0.32	0.40	0.34	1.0			0.36	0.47	0.42	1.0			0.35
4. PCONC	0.73	0.62	0.41	1.0		0.53	0.81	0.76	0.41	1.0		0.52	0.74	0.68	0.49	1.0		0.54
5. PCONT	0.34	0.34	0.73	0.28	1.0	0.58	0.30	0.29	0.79	0.33	1.0	0.62	0.37	0.36	0.76	0.35	1.0	0.62
TFF						0.48						0.55						0.55

All standardized factor loadings and correlations are significant at 0.05 level. MU, mutuality; COM, communication; HC, harmony and conflict; PCONC, parental concern; PCONT, parental control; TFF, total score of family functioning.

### Multigroup invariance across subsamples

A series of invariance tests were conducted across two subsamples at each wave to assess multigroup invariances of the C-FAI. As indicated in Table 2, the five-factor correlated model of the C-FAI showed an acceptable fit to the data of the subsamples at each wave, with NNFI and CFI values ranging from 0.93–0.96 and 0.94–0.97, respectively. As such, a series of factorial invariance tests were conducted across two subsamples in each wave of data subsequently. As the result of the chi-square difference test is too sensitive to large sample size (Schumacker and Lomax, 2004), a practical approach was adopted (ΔCFI ≤0.002; ΔRMSEA <0.01) for demonstrating the measurement invariance property of the C-FAI in the present study (Chen, 2007; Little, 2013). The findings of this study revealed equivalent fit indices between all pairs of the more restrictive model and the comparison model since changes in CFI and RMSEA values were less than the cutoff values of 0.002 and 0.01, respectively. As such, C-FAI is measurement and structural invariant across subsamples of three waves of data. Multigroup invariance of the C-FAI was confirmed and Hypothesis 2 was supported. Specifically, the most restrictive model of the C-FAI supposing equality in factor loadings, intercepts, uniqueness of indicators, factor variances and covariances illustrated fair fit indices at Wave 1 to Wave 3 (RMSEA and SRMR values ranged from 0.072–0.104, and 0.102–0.121, respectively), in spite of good fit demonstrated by NNFI and CFI indices.

**Table 2:** Multigroup invariance of the C-FAI across two subsamples at Wave 1, Wave 2, and Wave 3.

	Global fit indices							Models	Δ test			
	*SBχ^2^*	*df*	*p* value	*NNFI*	*CFI*	*RMSEA*	*SRMR*		*SBχ^2^*	*p* value	*CFI*	*RMSEA*
Invariance tests at Wave 1												
Sample 1 (*N* = 1863)	10169.4	481	<0.001	0.93	0.94	0.104	0.113	-	-	-	-	-
Sample 2 (*N* = 1867)	8640.2	481	<0.001	0.94	0.95	0.095	0.098	-	-	-	-	-
1.Configural MI	18266.4	962	<0.001	0.939	0.944	0.098	0.098					
2.Weak MI	18483.1	990	<0.001	0.940	0.944	0.097	0.102	1 vs. 2	216.7	<0.001	0.000	0.001
3.Strong MI	18725.1	1,023	<0.001	0.942	0.944	0.096	0.102	2 vs. 3	242.0	<0.001	0.000	0.001
4.Strict MI	18508.0	1,056	<0.001	0.944	0.944	0.094	0.101	3 vs. 4	217.1	<0.001	0.000	0.002
5.Factor variance MI	18516.2	1,061	<0.001	0.944	0.944	0.094	0.101	4 vs. 5	8.2	0.146	0.000	0.000
6.Factor covariance MI	18506.6	1,071	<0.001	0.945	0.944	0.093	0.102	5 vs. 6	9.6	0.476	0.000	0.001
Invariance tests at Wave 2												
Sample 1 (*N* = 1864)	4772.5	481	<0.001	0.96	0.97	0.069	0.104	-	-	-	-	-
Sample 2 (*N* = 1868)	5935.8	481	<0.001	0.96	0.96	0.078	0.102	-	-	-	-	-
1.Configural	10976.6	962	<0.001	0.960	0.964	0.075	0.102					
2.Weak MI	11214.1	990	<0.001	0.961	0.963	0.074	0.103	1 vs. 2	237.5	<0.001	0.001	0.001
3.Strong MI	11386.5	1,023	<0.001	0.962	0.963	0.074	0.103	2 vs. 3	172.4	<0.001	0.000	0.000
4.Strict MI	11281.3	1,056	<0.001	0.963	0.963	0.072	0.104	3 vs. 4	105.2	<0.001	0.000	0.002
5.Factor variance MI	11292.2	1,061	<0.001	0.963	0.963	0.072	0.104	4 vs. 5	10.9	0.053	0.000	0.000
6.Factor covariance MI	11339.0	1,071	<0.001	0.964	0.963	0.072	0.105	5 vs. 6	46.8	<0.001	0.000	0.000
Invariance tests at Wave 3												
Sample 1 (*N* = 1859)	10739.5	481	<0.001	0.94	0.95	0.107	0.126	-	-	-	-	-
Sample 2 (*N* = 1857)	10674.6	481	<0.001	0.94	0.94	0.107	0.120	-	-	-	-	-
1.Configural	22550.3	962	<0.001	0.940	0.945	0.110	0.120					
2.Weak MI	22748.7	990	<0.001	0.942	0.945	0.109	0.120	1 vs. 2	198.4	<0.001	0.000	0.001
3.Strong MI	23069.9	1,023	<0.001	0.943	0.945	0.108	0.120	2 vs. 3	321.2	<0.001	0.000	0.001
4.Strict MI	22573.3	1,056	<0.001	0.945	0.945	0.105	0.121	3 vs. 4	496.6	<0.001	0.000	0.003
5.Factor variance MI	22592.3	1,061	<0.001	0.945	0.945	0.105	0.121	4 vs. 5	19.0	<0.01	0.000	0.000
6.Factor covariance MI	22609.9	1,071	<0.001	0.946	0.945	0.104	0.121	5 vs. 6	17.6	0.062	0.000	0.001

NNFI, non-normed fit index; CFI, comparative fit index; RMSEA, root-mean-square error of approximation; SRMR, standardized root-mean square residual; MI, measurement invariance.

### Longitudinal invariance across time

After confirming the multigroup invariant property of the C-FAI, its longitudinal invariance was further explored. As the five-factor correlated model of the C-FAI demonstrates an acceptable fit to the data of each wave (see Table 1), a series of measurement invariance tests were conducted over three waves of data subsequently to investigate the longitudinal invariance of the C-FAI. In Table 3, Model 1 demonstrated a good fit to the observed data (χ^2^ = 58942.8, df = 4,536, *p* < 0.001, NNFI = 0.961, CFI = 0.964, RMSEA = 0.071, and SRMR = 0.073), suggesting the generalizability of the factor structure of the C-FAI over time (configural invariance). Then, a more restricted model (Model 2) for assessing the weak measurement invariance of the C-FAI was performed. In Model 2, factor loadings were specified to be the same across three waves of data. As the change in both CFI and RMSEA values between Model 1 and Model 2 were less than 0.002, the weak measurement invariance of the C-FAI was supported. Given all factor loadings of items were invariant, strong measurement invariance of the C-FAI was examined. In this form of invariance test, factor loadings and intercepts were specified to be equal across three waves of data in Model 3. Since there was no change in CFI and RMSEA values between Model 2 and Model 3, the strong measurement invariance of the C-FAI was also supported. Given all factor loadings and intercepts of items were invariant, strict measurement invariance of the C-FAI was examined. In this form of invariance test, factor loadings, intercepts as well as uniqueness of indicators were constrained to be identical across three waves of data (Model 4). As the change in CFI values between Model 3 and Model 4 was 0.003, which was greater than the cutoff value of 0.002, the strict measurement invariance of the C-FAI was not supported. In sum, the findings of this study indicate that the factor structure of the C-FAI remained consistent across time, demonstrating longitudinal invariance. Additionally, latent means could be compared without bias. This confirmed Hypothesis 3. In sum, C-FAI has good factorial validity and possesses multigroup and longitudinal invariant properties across sub-samples and over time.

**Table 3:** Longitudinal measurement invariance of the C-FAI across time (Wave 1 to Wave 3).

Invariance model	Global fit indices							Models	Δ test			
	*χ^2^*	*df*	*p* value	*NNFI*	*CFI*	*RMSEA*	*SRMR*		*χ^2^*	*p* value	*CFI*	*RMSEA*
1.Configural	58942.8	4,536	<0.001	0.961	0.964	0.071	0.073	-	-	-	-	-
2.Weak MI	59116.1	4,592	<0.001	0.961	0.963	0.071	0.073	1 vs. 2	173.3	<0.001	0.001	0.000
3.Strong MI	59974.7	4,658	<0.001	0.961	0.963	0.071	0.073	2 vs. 3	858.6	<0.001	0.000	0.000
4.Strict MI	63858.1	4,724	<0.001	0.959	0.960	0.074	0.074	3 vs. 4	3883.4	<0.001	0.003	0.003

NNFI, non-normed fit index; CFI, comparative fit index; RMSEA, root-mean-square error of approximation; SRMR, standardized root-mean square residual; MI, measurement invariance.

### Convergent and discriminant validity

Regarding convergent and discriminant validity of the C-FAI, the findings revealed that the average values of AVE for all factors across three waves (except the harmony and conflict factor) ranged from 0.53 to 0.61, which were higher than the cutoff value of 0.50 (Table 4). In addition, the total score of family functioning correlated significantly (*p* < 0.05) and substantially with three conceptually related indicators in each wave of data, including (1) there is mutual support among family members (r ranged from 0.34 to 0.35), (2) family members know to understand the situations of each other (all rs were 0.40), and (3) relationship between you and caregivers (r ranged from 0.43 to 0.52). As such, Hypothesis 4 was supported and the convergent validity of the C-FAI was demonstrated. Regarding discriminant validity of the C-FAI, the findings revealed that the total score of family functioning did not correlate substantially with other three conceptually unrelated indicators, including (4) amount of sleep per day (r ranged from 0.00 to 0.14), (5) amount of time for doing exercise per day (r ranged from −0.01 to 0.01), and (6) on average, the amount of sweet drink which you take per week/month (r ranged from −0.12 to −0.17). Consequently, Hypothesis 5 was supported and discriminant validity of the C-FAI was confirmed.

**Table 4:** Correlations between total score of family functioning and six indicators at three waves of data.

Indicators	Wave 1	Wave 2	Wave 3
1. Family members mutually support each other.	0.34	0.35	0.35
2. Family members know to understand the situations of each other.	0.40	0.40	0.40
3. Is the relationship between you and your caregivers good?	0.43	0.50	0.52
4. What is your daily amount of sleeping time?	0.14	0.10	(0.00)
5. What is your daily amount of time to do exercises?	(0.01)	(−0.01)	(−0.01)
6. On average, how much sweet drink do you take in per week / month?	−0.16	−0.12	−0.17

The correlation coefficient inside the bracket is insignificant (*p* > 0.05).

### Reliability

Table 5 illustrates the reliability of five subscales and the total scale of the C-FAI. The findings of this study showed that the composite reliability of the subscales and the total scale ranged from 0.72–0.97 at Wave 1, 0.74–0.97 at Wave 2, and 0.75–0.97 at Wave 3. They illustrated that the C-FAI was reliable. The reliability of the C-FAI was further supported by Cronbach’s alpha and mean inter-item correlations (at Wave 1: 0.67–0.94 and 0.28–0.57; at Wave 2: 0.62–0.95 and 0.33–0.61; at Wave 3: 0.66–0.95 and 0.32–0.62, respectively). As such, Hypothesis 6 was supported. In sum, the factorial, convergent and discriminant validity as well as the reliability of the C-FAI were confirmed. In addition, longitudinal and multigroup invariance of it were evident. As such, C-FAI is a psychometrically sound measure to investigate adolescents’ perceived family functioning in mainland China.

**Table 5:** Reliability of the C-FAI based on the whole sample at three waves of data.

	Mutuality	Communication	Harmony and conflict	Parental concern	Parental control	Total scale
Wave 1						
Composite reliability	0.93	0.90	0.72	0.75	0.80	0.97
α	0.93	0.91	0.69	0.67	0.80	0.94
Mean inter-item correlation	0.53	0.52	0.28	0.43	0.57	0.35
Wave 2						
Composite reliability	0.95	0.92	0.76	0.74	0.83	0.97
α	0.95	0.93	0.75	0.62	0.83	0.95
Mean inter-item correlation	0.61	0.59	0.33	0.41	0.61	0.39
Wave 3						
Composite reliability	0.95	0.92	0.76	0.75	0.83	0.97
α	0.95	0.93	0.74	0.66	0.83	0.95
Mean inter-item correlation	0.62	0.60	0.32	0.43	0.62	0.41

## Discussion

This study aimed to examine the factor structure, convergent validity, discriminant validity and reliability of the C-FAI as well as its invariance across subsamples and time, among children and adolescents residing in mainland China. One of the primary features of this work was its use of a longitudinal research approach with a large sample size to perform a construct validation study of the C-FAI. As such, the stability of the factor structure of the C-FAI over time was explored. It is important because the longitudinal invariant property of the C-FAI is largely neglected in previous studies using family functioning measures (e.g., Wang et al., 2021, 2023; Wang X. et al., 2022; Zhang et al., 2023). Besides, apart from adopting RML estimation to address multivariate non-normality of the data, this study recruited a large sample for investigation. This would lower standard errors of the estimates and hence, enhance the accuracy and credibility of the findings. In addition, as most of the self-reported family functioning measures focus on adults instead of children and adolescents (Tiffin et al., 2011), this study provided empirical support for the usefulness of the C-FAI to assess subjective family functioning among preadolescents and adolescents in mainland China.

In response to the question about the factor structure of the C-FAI among preadolescents and adolescents in mainland China (Research Question 1), our findings offered empirical support for the five-factor structure of the C-FAI (mutuality, communication, conflict and harmony, parental concern, and parental control), hence supporting Hypothesis 1. It echoes the findings of Siu and Shek’s (2005) and Shek and Ma’s (2010) study, which revealed the same factor structure of the C-FAI among adolescents in Hong Kong. As stated by Cultural Atlas Editors (2016), even though there are some social and cultural differences between people in Hong Kong and mainland China, Confucianism still serves as the foundation of the cultural roots of people in both places. Some important Confucian values, such as filial piety, are still prevalent among children and adolescents, which in turn determine their perceptions of a good and healthy family (Li et al., 2014). For instance, under the filial piety tradition, children and adolescents will follow and respect their parents while parents will take care of and accept their children. As such, family harmony would be preserved by developing mutuality among family members. Moreover, as the dimensions of the C-FAI identified in this study have high similarity to three important dimensions of family functioning in Western studies, which are cohesiveness, communication, and flexibility, these three aspects of family functioning seem to be universal across both Western and Chinese cultural contexts.

Nonetheless, similar to the findings using Hong Kong adolescents (Siu and Shek, 2005), the “conflict and harmony” dimension identified in this study reflects that the “absence of conflict” is also viewed as an important element of a good family for children and adolescents in mainland China. In addition, “parental concern” and “parental control” factors are associated with the functioning of parents, which in turn reflects the significant role of parents in determining the functioning of families in mainland China. In sum, as stated by Wong et al. (2022), there are two perspectives to conceptualize family functioning, including process-oriented and result-oriented perspectives. The former perspective classifies families into different kinds based on the features of the family (e.g., see Olson’s (2000) Annular Mode model of family functioning), while the latter perspective is mainly concerned with the essential components for the development of healthy families (e.g., see Miller et al.’s (2000) McMaster family functioning model). The dimensions of the C-FAI involve both result-oriented (mutuality, communication, and conflict and harmony) and process-oriented elements (parental concern and parental control), which would offer holistic insights into the development of positive family functioning in mainland China.

For the second research question, our findings supported Hypothesis 2 that the dimensions underlying the C-FAI were invariant across random subsamples. It is consistent with the findings of Shek and Ma’s (2010) study, which illustrated strong measurement invariance of the C-FAI across subsamples based on case numbers (even and odd). However, the results of this study offered additional empirical evidence in support of the strict measurement invariance and structural invariance of the C-FAI across random subsamples. These findings suggest two random subsamples have same interpretations of C-FAI items, and the factor and observable means of the level of family functioning between two subsamples could be compared without bias. Moreover, the relationships among the five factors of the C-FAI were equally applied to two subsamples. As such, two subsamples have the same conceptual understanding of the areas of functioning in Chinese families. For example, the “mutuality” and “communication” factors would be highly correlated because effective communication among family members would promote their mutuality (White et al., 2010).

The findings of this study indicate that the factor structure of the C-FAI remained consistent throughout time, hence providing support for Hypothesis 3. This finding provides evidence for the long-term stability of the C-FAI. Please be advised that the use of C-FAI has been observed in longitudinal research conducted with teenagers in mainland China (e.g., Wang et al., 2021, 2023), the longitudinal invariant property of the C-FAI has not been well addressed. The strict longitudinal invariance of the C-FAI found in this study adds to the extant literature and supports the fact that the C-FAI assesses the same family functioning construct at different points of time. As such, C-FAI could be used to assess age-varying changes in the subjective family functioning of Chinese people from childhood to adolescence to adulthood, especially during the period of adolescence in which teenagers may have negative relationships with parents (Gniewosz and Gniewosz, 2020). In addition, as mentioned in the review article by Dai and Wang (2015), research on the development of family functions at different periods during the life of a family is very limited. C-FAI would be a promising family functioning measure to address this gap and assess changes in the functions of a family over the life course.

Apart from supporting the factorial validity of the C-FAI, the construct validity of the C-FAI was further confirmed by establishing its convergent and discriminant validity. Regarding the fourth research question, the present results revealed substantial correlations between the total score of the C-FAI and the measures of family support, hence supporting Hypothesis 4. Convergent validity of the C-FAI was confirmed. The results of Gaspar et al.’s (2022) research align with the findings presented here, demonstrating a significant positive association between parental emotional support and family functioning among a sample of 1,757 parents from Portugal. ‘Besides, the present results did not reveal substantial associations between the total score of C-FAI and theoretically unrelated constructs, and thus supported Hypothesis 5. As such, discriminant validity of the C-FAI was also confirmed. As stated by Strauss and Smith (2009), construct validity of a measure is commonly regarded as a unifying form of validity for psychological measurements and hence encompasses cumulative sources of evidence supporting specific interpretations of a score from a measure. The establishment of the convergent validity and discriminant validity of the C-FAI definitely offers additional validity support for what the C-FAI intends to measure, that is perceived family functioning.

Lastly, the findings of this study illustrated acceptable reliability of the total and subscale measures of the C-FAI, hence supporting Hypothesis 6. It is consistent with the findings of Hu et al.’s (2023) and Lam and Chen’s (2022) study, which showed the total scale of the C-FAI and its subscales were reliable. In sum, C-FAI is a valid and reliable measure of perceived family functioning among children and adolescents in mainland China. It is a stable family functioning instrument that would be utilized to compare the latent means between groups and detect the changes of latent means across time.

## Implications

Theoretically, this study provided empirical support to an indigenous conceptualization of family functioning in the Chinese context. As stated by Dai and Wang (2015), theoretical models of family functioning in China are mainly focused on translated literature and the Western-developed models may not be culturally appropriate in the Chinese culture. Therefore, the development of unique Chinese family functioning models is of paramount importance. Hence, the study is an innovative attempt using rigorous conceptual arguments and research methods (e.g., longitudinal design and use of confirmatory factor analyses). This study also paves the way for the development of more sophisticated family functioning models for Chinese people.

Basically, family functioning theory is classified into two categories in the West. The first one is result-oriented family functioning theory, which defines family functioning by special features of the family such as family intimacy and family communication styles. Another one is process-oriented family functioning theory, which describes family functioning in terms of tasks families need to complete, such as affective involvement and behavior control of the child in the family. Literature review has illustrated that a theoretical model of family functioning with both result-and process-oriented elements is very rare. However, the conceptual model underlying the C-FAI is composed of both result-oriented elements (mutuality, communication, and conflict and harmony) and process-oriented ones (parental concern and parental control). This conceptual model of family functioning would add to the literature and serve as an innovative reference model to facilitate the cross-cultural examination of family functioning in different cultural contexts.

Practically, C-FAI would serve as a psychometrically sound family functioning instrument to identify family problems and hence support clinical practices in mainland China and Hong Kong. In light of the increase in family problems, youth education problems and psychological problems in mainland China in recent years, the demand for family therapy and intervention has been raised by leaps and bounds (Yao, 2022). As such, the provision of family therapy and intervention has been greatly increased. Nevertheless, Quek and Chen (2017) commented on the applicability of Western-based family therapy approaches and screening instruments to the Chinese context. As C-FAI has been developed in the Chinese context, it could be utilized to conduct family functioning research in mainland China and Hong Kong appropriately. In fact, Hu et al. (2023) have already utilized the C-FAI to identify families with different levels of family environment dysfunction and subsequently explored the effects of the family environment on non-suicidal self-injury among secondary school students in mainland China. C-FAI would be used to help counselors and family therapists to identify the problematic areas of family functioning in an unhealthy family and subsequently provide appropriate intervention and treatment to clients. In addition, as the current findings illustrated that the C-FAI exhibited favorable psychometric properties, it could be utilized as an objective reference tool in future studies on family functioning within various Asian contexts, thereby contributing to the broader international research landscape.

## Limitations

There are certain limitations of the study. First, we only used three-wave data to assess longitudinal invariance of the C-FAI. To delineate a holistic picture of measurement invariant property of the C-FAI over time, future research should aim to collect more waves of data over an extended period of time. Second, multigroup invariance of the C-FAI was assessed using random subsamples only. As family functioning has been found to be associated with gender and family SES of the participants (Berge et al., 2013), future research should explore whether the C-FAI is invariant across gender and family SES among children and adolescents in mainland China. Third, the study sample was limited to preadolescents and adolescents residing in Chengdu. Although studies focusing on a single province have been conducted (e.g., Dou et al., 2021; Wang L. et al., 2022), it is necessary to replicate the generalizability of the current findings across diverse populations in various regions of China.

## Conclusion

This innovative study aimed to examine the factorial validity, convergent validity, discriminant validity, reliability and measurement invariance of the C-FAI in preadolescents and adolescents in mainland China. Based on rigorous conceptual arguments and utilizing advanced research design and methods, the results of this study provided support for all kinds of validity of the C-FAI and its multigroup and longitudinal invariance. As such, we conclude that C-FAI is a valid and reliable tool to assess perceived family functioning among children and adolescents in mainland China. The present findings provide support for an integrated indigenous Chinese model of family functioning. Besides, in view of its sound psychometric properties, the practical significance of the findings is that family practitioners and researchers can utilize the C-FAI to identify different problematic areas of the functioning in Chinese families and implement effective intervention and treatment to their clients.

## Data availability statement

The raw data supporting the conclusions of this article will be made available by the authors, without undue reservation.

## Ethics statement

The studies involving humans were approved by Ethics Research Committee of Sichuan University. The studies were conducted in accordance with the local legislation and institutional requirements. Written informed consent for participation in this study was provided by the participants’ legal guardians/next of kin.

## Author contributions

DS: Conceptualization, Funding acquisition, Investigation, Methodology, Supervision, Writing – review & editing. KL: Formal analysis, Writing – original draft. XL: Methodology, Writing – review & editing. DD: Writing – review & editing.
